# Drawbacks of Low Lattice Energy Ammonium Salts for Ion-Conducting Polymer Electrolyte Preparation: Structural, Morphological and Electrical Characteristics of CS:PEO:NH_4_BF_4_-Based Polymer Blend Electrolytes

**DOI:** 10.3390/polym12091885

**Published:** 2020-08-21

**Authors:** Mohamad A. Brza, Shujahadeen B. Aziz, Muaffaq M. Nofal, Salah R. Saeed, Shakhawan Al-Zangana, Wrya O. Karim, Sarkawt A. Hussen, Rebar T. Abdulwahid, Mohd F. Z. Kadir

**Affiliations:** 1 Manufacturing and Materials Engineering Department, Faculty of Engineering, International Islamic University of Malaysia, Kuala Lumpur 50603, Gombak, Malaysia; mohamad.brza@gmail.com; 2 Hameed Majid Advanced Polymeric Materials Research Lab., Department of Physics, College of Science, University of Sulaimani, Sulaimani 46001, Kurdistan Regional Government, Iraq; sarkawt.hussen@univsul.edu.iq (S.A.H.); rebar.abdulwahid@univsul.edu.iq (R.T.A.); 3 Department of Civil Engineering, College of Engineering, Komar University of Science and Technology, Sulaimani 46001, Kurdistan Regional Government, Iraq; 4 Department of Mathematics and General Sciences, Prince Sultan University, P.O. Box 66833, Riyadh 11586, Saudi Arabia; muaffaqnofal@gmail.com; 5 Charmo Research Center, Charmo University, Peshawa Street, Chamchamal, Sulaimani 46001, Kurdistan Regional Government, Iraq; salah.saeed@charmouniversity.org; 6 Department of Physics, College of Education, University of Garmian, Kalar 46021, Kurdistan Regional Government, Iraq; shakhawan.al-zangana@garmian.edu.krd; 7 Department of Chemistry, College of Science, University of Sulaimani, Qlyasan Street, Sulaimani 46001, Kurdistan Regional Government, Iraq; wrya.karim@univsul.edu.iq; 8 Department of Physics, College of Education, University of Sulaimani, Old Campus, Sulaimani 46001, Kurdistan Regional Government, Iraq; 9 Centre for Foundation Studies in Science, University of Malaya, Kuala Lumpur 50603, Malaysia; mfzkadir@um.edu.my

**Keywords:** polymer blend electrolyte, low lattice energy ammonium salts, degree of crystallinity, morphology study, impedance spectroscopy, dielectric properties

## Abstract

In the present work it was shown that low lattice energy ammonium salts are not favorable for polymer electrolyte preparation for electrochemical device applications. Polymer blend electrolytes based on chitosan:poly(ethylene oxide) (CS:PEO) incorporated with various amounts of low lattice energy NH_4_BF_4_ammonium salt have been prepared using the solution cast technique. Both structural and morphological studies were carried out to understand the phenomenon of ion association. Sharp peaks appeared in X-ray diffraction (XRD) spectra of the samples with high salt concentration. The degree of crystallinity increased from 8.52 to 65.84 as the salt concentration increased up to 40 wt.%. These are correlated to the leakage of the associated anions and cations of the salt to the surface of the polymer. The structural behaviors were further confirmed by morphological study. The morphological results revealed the large-sized protruded salts at high salt concentration. Based on lattice energy of salts, the phenomena of salt leakage were interpreted. Ammonium salts with lattice energy lower than 600 kJ/mol are not preferred for polymer electrolyte preparation due to the significant tendency of ion association among cations and anions. Electrical impedance spectroscopy was used to estimate the conductivity of the samples. It was found that the bulk resistance increased from 1.1 × 10^4^ ohm to 0.7 × 10^5^ ohm when the salt concentration raised from 20 wt.% to 40 wt.%, respectively; due to the association of cations and anions. The low value of direct current (DC) conductivity (7.93 × 10^−7^ S/cm) addressed the non-suitability of the electrolytes for electrochemical device applications. The calculated values of the capacitance over the interfaces of electrodes-electrolytes (C_2_) were found to drop from 1.32 × 10^−6^ F to 3.13 × 10^−7^ F with increasing salt concentration. The large values of dielectric constant at low frequencies are correlated to the electrode polarization phenomena while their decrements with rising frequency are attributed to the lag of ion polarization in respect of the fast orientation of the applied alternating current (AC) field. The imaginary part of the electric modulus shows obvious peaks known as conduction relaxation peaks.

## 1. Introduction

Polymer electrolytes are considered a significant category of solid-state co-ordination compounds that are essential for ionic conductivity in solid and flexible membranes [1,2]. The field of ion conducting solid polymer electrolytes (SPEs) was invented by Michel Armand in 1972 [3,4]. The application of SPEs for battery technology over liquid electrolytes is well documented in the literature [4,5,6]. Compared to liquid electrolytes, these SPEs depict better mechanical properties, convenient manufacturing of thin films, appropriate sizes, and formation of appropriate contact between the electrode and the electrolyte [7]. Chain polymers having an electro-negative atom (oxygen or nitrogen) within their repeated unit were also found to serve as a good solvent for particular salts due to the interaction between chains and cations. The presence of both atoms in chitosan (CS) polymer was revealed in various past research works [1,2,7,8]. Peter V. Wright and Bruceet. al., established that complexation involves an interaction of the cation with the lone pairs of the ether oxygen atoms existing in a poly(ethylene oxide) (PEO)-based polymer electrolyte [9,10]. The shells of crab and shrimp are the main sources of chitin from which CS is derived. During the deacetylation of chitin, CS is produced to overcome the limited solubility of chitin in common solvents. The existence of the functional groups of NH_2_ and OH in CS act as conjunction sites and allows CS to be a better sorbent characterized with greater affinity for transition metal ions. Other properties of CS include capability of film-formation, porous scaffolds, as well as hydrogels [11,12,13,14].

Polymer electrolytes are among the significant materials in the research field and play an important role in progression of electrochemical devices. The main subject of the majority of research in this field is solid state electrochemistry, which focused on the development of high ion-conducting materials for various applications related to energy conversion and storage [15,16]. It can be found in the literature that many ion-conducting polymer electrolytes were developed on the basis of CS and its blends [17,18,19,20,21]. Various polymers for example poly(vinylidene fluoride) (PVDF)[22], polyvinyl pyrrolidone (PVP) [23], boroxine ring polymer (BP) [24], polymethylhydrogen-siloxane (PMHS) [25], poly(vinyl alcohol) (PVA) [26] as well as poly(dithiooxamide) (PDTOA) [27] were mixed with PEO to fabricate polymer electrolytes. PEO polymer possesses a small melting point [28] and, therefore, its mixing with large glass transition temperature polymers for example CS is vital for constructing flexible films. In addition, the biodegradability and environmental-friendly properties of these polymers encouraged the researchers to put more efforts in employing these polymers in energy devices so as to reduce the released toxic and hazardous materials [29,30,31,32]. On the basis of early studies [19,28], it was demonstrated that the optimal ratio is 70 wt.% CS and 30 wt.% PEO is adequate to construct films with a large amorphous structure. Similarly, in the present work, 70 wt.% CS-30 wt.% PEO is selected with different amount of ammonium tetrafluoroborate (NH_4_BF_4_). 

It is well known that ions required for the conduction process are provided by the added salts and the polymer electrolyte conductivity is also affected by the lattice energy of salts [21,33]. It was previously demonstrated that CS reduced transition metal salts for example copper or silver salts [21,34]. This is because the functional groups of NH_2_ and OH along the chains of the polymer reduce ions of copper or silver to their metallic nano-particles [34,35,36]. In our early studies we have demonstrated that CS-based electrolytes inserted with the transition metal salts are not appropriate for applications in electrochemical devices due to the reduction of copper or silver ions to metallic particles and hence are missing great quantities of conducting ions [21,34,35,36]. Earlier works established that the properties of ion-conducting polymer electrolytes play the key role in determining the performance of electrochemical devices [37,38,39,40]. It was known that, for the applications in electrochemical devices, the films conductivity must be at least in the range between 10^−5^ and 10^−3^ S/cm [41,42,43]. However, the polymer electrolyte with the conductivity of 10^−5^ S/cm has been applied for electric double-layer capacitors (EDLC) in our previous work, and it exhibited very low performance [44].

The performance of electrochemical and energy devices is significantly impacted by both structural and electrical properties of the used polymer electrolyte [45,46]. Therefore, careful selections of the host polymer with a suitable salt are the key factors in the preparation of the polymer electrolyte for practical applications. This work aims to highlight the effect of the lattice energy of salt on the structural, morphological and electrical properties of polymer electrolyte. In this research polymer blends of CS:PEO are doped with various concentration of NH_4_BF_4_ salt and the effects of salt lattice energy on the characteristics of the prepared blended polymer electrolyte are thoroughly investigated. For this purpose, the electrochemical impedance spectroscopy (EIS) that is directly linked to electrochemistry will be employed, and then the link between direct current (DC) conductivity and ion association can be demonstrated. EIS of polymers are an influential implement to discover the structural creation and produce helpful complementary indication for the electrical properties of polymer materials [47]. To support our electrochemical impedance results, various techniques, such as scanning electron microscopy (SEM) and X-ray diffraction (XRD) will be also used. Furthermore, dielectric characteristics, which are the main comprehensively investigated subjects in condensed matter physics, along with structural, morphological as well as impedance examination will be investigated [48]. The dielectric relaxation examination in polymer electrolytes offers a superior perceptive on the characteristics of the reaction among cations and polymers; furthermore, the salt dissolving characteristic of the polymer is demonstrated through the dielectric constant [49]. This work will provide a clear insight into the effects of salt lattice energy on the properties of the prepared polymer electrolyte, which is less addressed in the literature. The results of the present work demonstrate that ammonium salts with low lattice energy below around 600 kJ/mol are not desired for the fabrication of polymer electrolyte membranes. The samples of the present work exhibits low value of DC conductivity and dielectric constant. It is clear that based on the DC conductivity outcome, suitable electrolyte candidates can be chosen for a preferred application. Therefore, it is not possible for us to use our samples in the applications due to the occurrence of ion association.

## 2. Experimental Detail

### 2.1. Sample Preparation

Sigma-Aldrich (Kuala Lumpur, Malaysia) was approached in order to purchase the materials including CS having large molecular mass with the average molecular weight of 310,000–375,000 and poly (ethylene oxide) (PEO) powder with the average molecular weight of 300,000 to be used as raw materials. The polymer blend based on CS:PEO was prepared by taking 50 mL of 1% acetic acid as solvent and separately dissolving 70 wt.% CS and 30 wt.% PEO at room temperature for 2 h. This was followed by obtaining a homogeneous solution by mixing and stirring the solution for 3 h. Similarly, the homogenous solution of CS:PEO was obtained by constantly stirring the mixture and adding NH_4_BF_4_ in different proportions of weight (from 10% to 40 wt.%) in multiples of 10, yielding CS:PEO:NH_4_BF_4_ electrolytes. The polymer blend electrolytes were coded as CSPEH1, CSPEH2, CSPEH3, and CSPEH4 for CS:PEO doped with 10, 20, 30, and 40 wt.% of NH_4_BF_4_, correspondingly. Then, the casting process was started in various Petri dishes, and these solutions were kept for drying at surrounding temperature for the films to form. Lastly, the films were kept in a desiccator to dry further, creating films without solvent. 

### 2.2. Structural, Morphological and Impedance Characterizations

A D5000 X-ray diffractometer (1.5406 Å) was used to record the XRD pattern and conduct structural analysis. Constant scanning of the 2θ angle from 10° to 80° (resolution = 0.1°) was performed during the acquisition process. The Hitachi SU8220 field-emission scanning electron microscope (FESEM) having 500× magnification was employed to study the electrolyte surface. Moreover, the samples’ electrical impedance spectra were studied using HIOKI 3532-50 LCR HiTESTER (50 Hz to 5 MHz) (Hioki, Nagano, Japan). The constructed films were reduced to small circles with diameter of 2 cm and located between stainless steel electrodes due to pressure of a spring. The cell is connected with a computer program to provide real part (Z’) and imaginary part (Z”) of the complex impedance (Z*) spectra.

## 3. Results and Discussion

### 3.1. Structural Study

**Figure 1:**
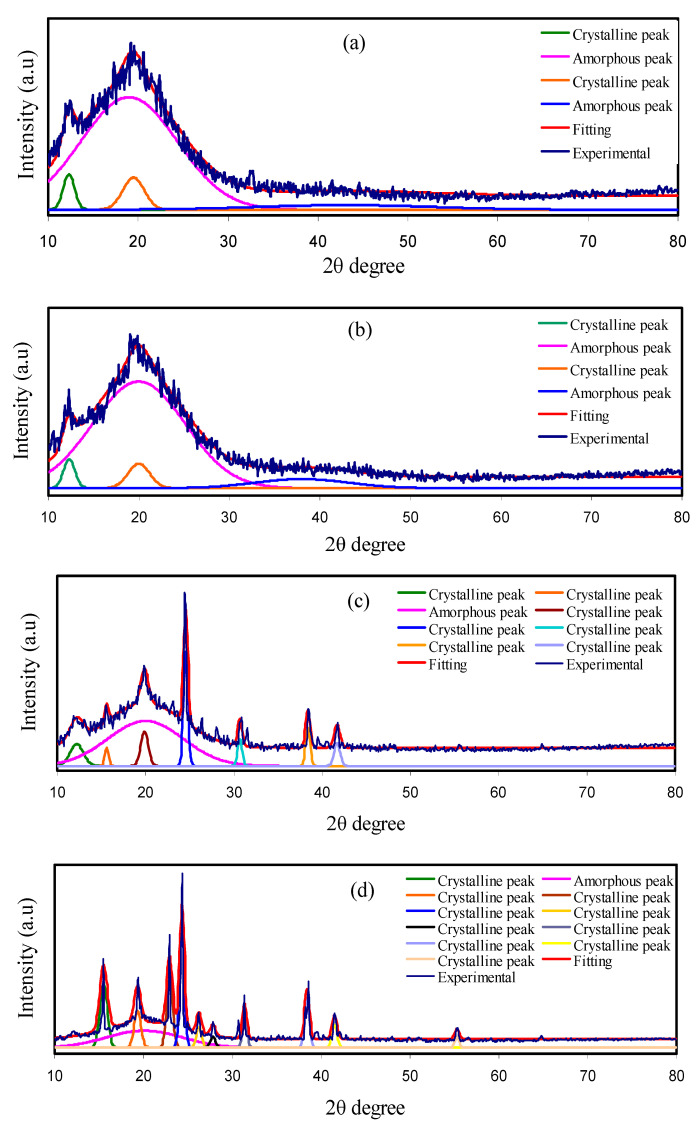
X-ray diffraction (XRD) pattern for (**a**) CSPEH1, (**b**) CSPEH2, (**c**) CSPEH3 and (**d**) CSPEH4 polymer blend electrolytes.

The details of the film structure can be explored with the help of the XRD technique. Figure 1a–d show the XRD patterns associated with CS:PEO films doped with various proportions of NH_4_BF_4_. Semi-crystalline composition of the pure CS and pure PEO were verified in various previous studies [28,50,51], along with their amorphous blending [19]. CS is found to form different crystalline peaks near 2θ = 15° and 20° which are principally owing to the crystalline component of the CS membrane’s average intermolecular distance [52,53]. The inter-molecular and intra-molecular hydrogen bonds, which are formed between the amino and hydroxyl groups through an absorbed water molecule, give a rigid crystalline nature to the CS [54,55]. One of the new techniques to disrupt intermolecular hydrogen bonds is the blending of polymers. Hence, the disruption of intermolecular hydrogen bonds and the polymer blending is depicted by the absence of crystalline peaks of CS as well as PEO considering the CS:PEO blend. The blended films of CS:PEO subjected to 10 and 20 wt.% doping show a decline in the intensity of peaks (see Figure 1a,b). However, when 30 and 40 wt.% of NH_4_BF_4_ were added to the blended films of CS:PEO, more crystalline and high intensity peaks have emerged in the XRD spectra (see Figure 1c,d). The formation of these peaks may be attributed to the association between the cation and anion of the salt and their leakage to the surface of the polymer. Conversely, the sample doped with 10 to 20 wt.% of the salt depicts the amorphous nature along with some low-intensity crystalline peaks [56]. These new peaks are due to the short-range order which is a consequence of the presence of the multiples ions [57]. Sanders et al. [58] considered these new peaks to be originated from the complex formation between polymer and salt instead of pure salt. 

The method of deconvolution for the XRD spectra has been applied in an attempt to acquire the likely crystalline peaks and amorphous peaks [59]. The degree of crystallinity (*X_c_*) has been achieved by means of the deconvoluted XRD spectra as observed in Figure 1. The wide and big peaks stand for the amorphous peaks; while, the crystalline peaks are presented as a narrow, sharp, and high intense peaks. The *X_c_* for CSPEH1 is 8.52 and notably reduced upon the insertion of 20 wt.% of NH_4_BF_4_. Hence, the amorphous nature of the system of polymer and salt is developed with the insertion of 20 wt.% of NH_4_BF_4_. The 30 and 40 wt.% of NH_4_BF_4_ caused more crystalline and high-intensity peaks as indicated in the XRD spectra of CSPEH3 and CSPEH4 (see Figure 1c,d). The *X_c_* for polymer electrolytes have been achieved by means of Equation (1) and listed in Table 1. The smallest *X_c_* that is 7.43 was achieved for CSPEH2. This means that CSPEH2 is the most amorphous polymer electrolyte in the current study. The DC ionic conductivity values obey the *X_c_* trend [60].

(1) $$X_{C}=\frac{A_{C}}{A_{T}}\times 100\%$$

where *A_C_* and *A_T_* stand for the total crystalline peaks areas, and total amorphous and crystalline peaks areas, respectively that have been attained by way of the deconvolution approach via the software of OriginPro. The mode of Gaussian function has been applied to fit the XRD spectra.

### 3.2. Morphological Study

**Figure 2:**
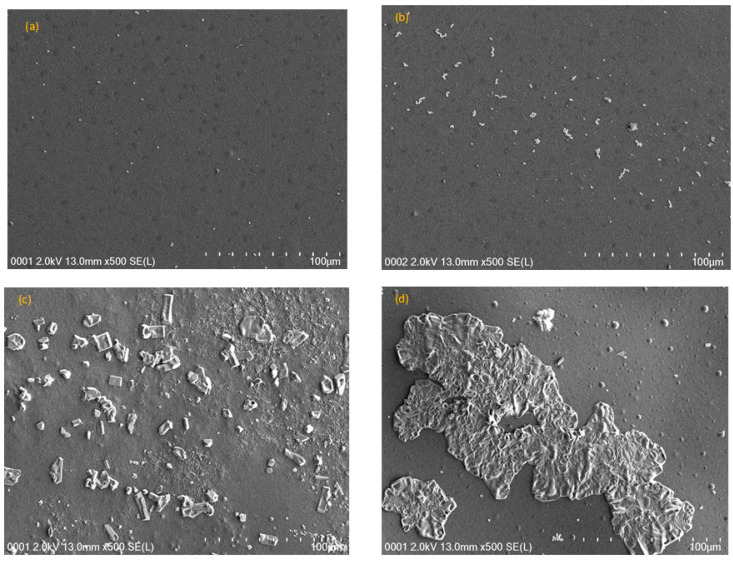
Field-emission scanning electron microscopy (FESEM) images for (**a**) CSPEH1, (**b**) CSPEH2, (**c**) CSPEH3 and (**d**) CSPEH4 polymer blend electrolytes.

**Figure 3:**
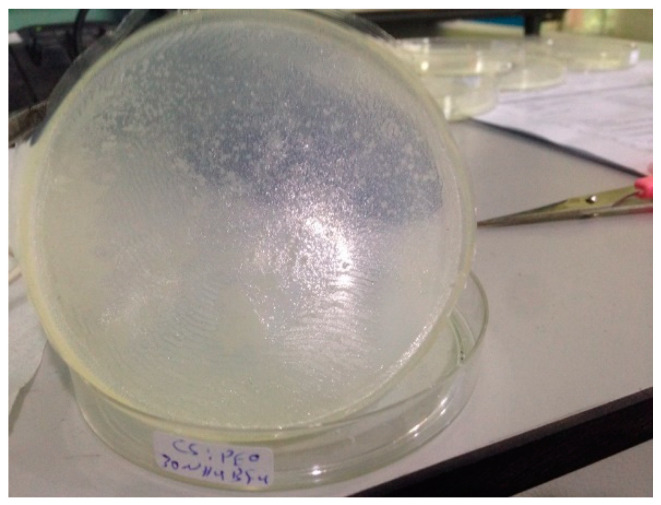
Realistic image of the prepared solid polymer electrolyte of CSPEH3 sample.

The SEM images were obtained to support the structural and surface study of the samples as shown in Figure 2. It is clear that the behavior of the polymer electrolyte films and blends can be determined with the help of their surface morphology and structure [61]. The image of the electron was taken after adjusting the magnification to 500×. A conductive tape was used to fix the films with the aluminum holder before examining the samples. The surfaces of the films doped with 10 wt.% and 20 wt.% of the salt revealed some small white spots (see Figure 2a,b). The protruded salt caused these white spots. Hence, there was correspondence between SEM images and XRD outcomes (see Figure 1). The images clearly show that the white spots increased by raising the salt concentration to 30 and 40 wt.% (see Figure 2c,d). It is clear from Figure 3, that 30 wt.% of NH_4_BF_4_ resulted in the salt to protrude through the surface of the electrolyte. Kadir et al. also obtained similar results using the SEM technique [62]. It is a known fact that the amorphous phase imparts smooth morphology form to the polymer blend electrolyte complex [42]. The SEM technique has been employed in our previous work to identify the crystalline structures ascribed to the creation of ion pairs in methyl cellulose (MC) based polymer electrolyte at high salt concentration [59]. The decline in DC conductivity in a CS:PEO electrolyte system with high NH_4_BF_4_ salt concentration is justified by the SEM outcomes. SEM was also employed by Kadir et al., for the detection of protruded crystalline salt structures in the CS based solid polymer electrolytes when salt concentrations were high [62,63]. The feasibility of employing the SEM method for the detection of protruded salt polymer blend electrolytes is also suggested by the outcomes of the current study. The presence of immense phase separation indicates more crystalline phases as similar results were obtained from the XRD study.

### 3.3. Impedance Analysis

**Figure 4:**
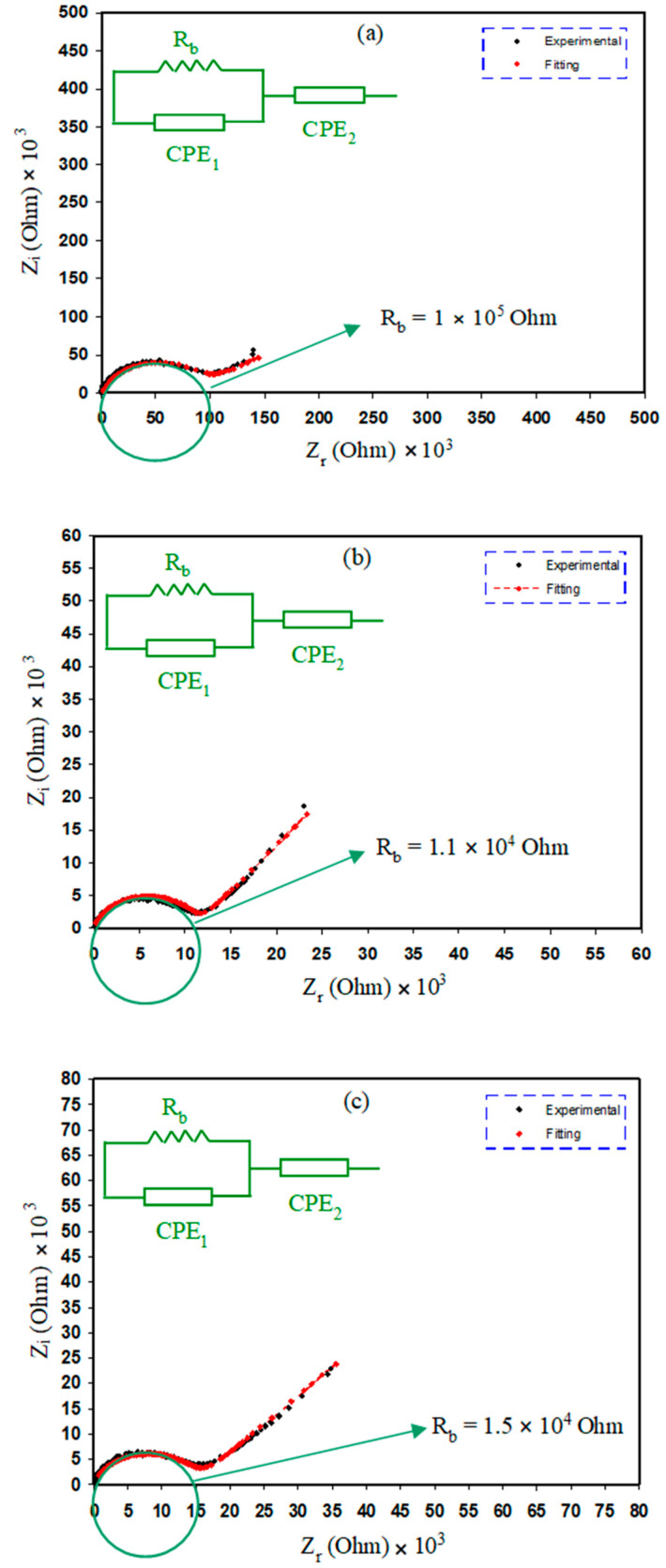

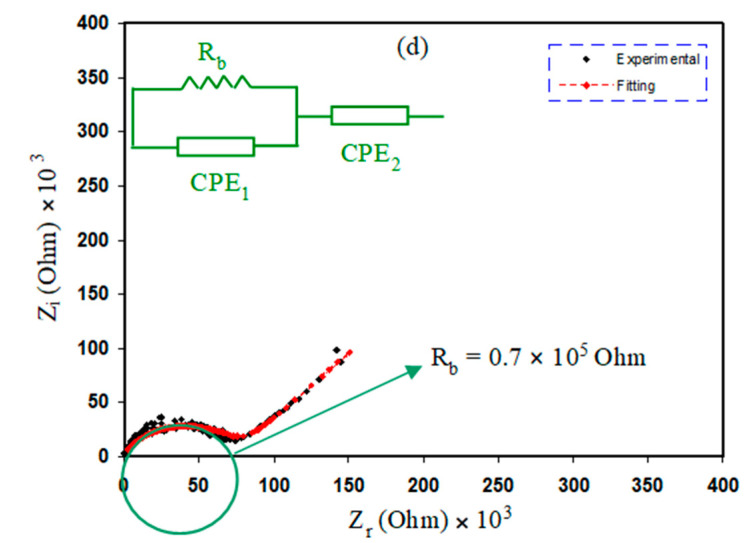
Electrical impedance plots for (**a**) CSPEH1, (**b**) CSPEH2, (**c**) CSPEH3 and (**d**) CSPEH4 polymer blend electrolytes.

The impedance spectrum for each of the blended electrolyte samples is depicted in Figure 4a–d. Separate regions for low and high frequencies are evident. It is essential to comprehend the process of charge transfer in the polymers from both fundamental and technological perspectives. The ionic conductivity of the polymeric materials and the charge transport mechanism of the complex materials can be investigated effectively and realized using impedance measurement technique [36,64]. The AC complex impedance spectroscopy involves the estimation of the impedance/admittance of the cell over various frequencies. Impedance considers phase difference in a distinct manner and it is a more detailed concept than the resistance. Considering alternating current (AC), instead of resistance, the impedance (Z) is used which is calculated by adding the resistance to the reactance [65]. The movement of ions via the electrolyte or the ionic conductivity is illustrated by a semicircle attained at the region of higher frequency [66]. The midpoint of the semicircle lies beneath the *x*-axis which shows the distribution of relaxation time [67,68]. Electrode polarization (EP) gives a straight line at low frequency over some data points [69]. EP is the consequence of the electric double-layer capacitances which emerge due to the accumulation of free charge on the electrolyte and electrode interfaces [70]. At the low-frequency region, the complex impedance graph must be a straight line parallel to the imaginary axis. This implies that if the straight line shows an inclination of 90°, the inclination is formed by the blocking electric double-layer capacitance at the electrodes [71,72].

The electrical equivalent circuit (EEC) routine has been completed to inspect the EIS since this method is uncomplicated, rapid, and gives a general picture of polymer electrolytes [73]. The Nyquist plots for the electrolytes were gained in respect to the equivalent circuit (EC) that contains R_b_ for the charge carriers in the electrolyte systems and two constant phase elements, i.e., CPE_1_ and CPE_2_ which are indicated in the Figure 4a–d inserts. The R_b_ and CPE_1_ combination are observed in parallel at the area of large frequencies. The CPE_2_ is demonstrated at the area of low frequencies where the double-layer capacitance between electrodes and polymer electrolytes can be constructed. Z_CPE_ impedance is indicated as [28,60]:

(2) $$Z_{\mathrm{CPE}}=\frac{1}{{C\omega }^{p}}\left[\cos\left(\frac{\pi p}{2}\right)-i\sin\left(\frac{\pi p}{2}\right)\right]$$

here, C is the CPE capacitance, ω is the angular frequency and P is connected to the EIS departure from the axes. The real and imaginary parts (*Z_r_* and *Z_i_*) of complex impedance (Z*) linked to the EC (insert of Figure 4a–d) are demonstrated as:

(3) $$Z_{r}=\frac{R_{b}{}^{2}C_{1}\omega^{P_{1}}\cos\left(\frac{\pi P_{1}}{2}\right)+R_{b}}{2R_{b}C_{1}\omega^{P_{1}}\cos\left(\frac{\pi P_{1}}{2}\right)+R_{b}{}^{2}C_{1}{}^{2}\omega^{2P_{1}}+1}+\frac{\cos\left(\frac{\pi P_{2}}{2}\right)}{C_{2}\omega^{P_{2}}}$$

(4) $$Z_{i}=\frac{R_{b}{}^{2}C_{1}\omega^{P_{1}}\sin\left(\frac{\pi P_{1}}{2}\right)}{2R_{b}C_{1}\omega^{P_{1}}\cos\left(\frac{\pi P_{1}}{2}\right)+R_{b}{}^{2}C_{1}{}^{2}\omega^{2P_{1}}+1}+\frac{\sin\left(\frac{\pi P_{2}}{2}\right)}{C_{2}\omega^{P_{2}}}$$

here, *C*_1_ is the capacitance of CPE_1_ at the polymer electrolytes bulk; *C*_2_ is the capacitance of CPE_2_ over the interfaces of electrodes-electrolytes; *P*_2_ implies the tail divergence from the *x*-axis, and *P*_1_ implies the semicircle radius divergence from the *y*-axis. The EEC fitting parameters are arranged in Table 2.

It is obvious that the bulk resistance (*R_b_*) (see the insets) reduces with the increment of salt concentration from 10 to 20 wt.%, but the bulk resistance experiences an escalation when the concentration of NH_4_BF_4_ salt is raised from 30 to 40 wt.%. The value of *R_b_* can be computed by identification of the point of intersection of the semicircle and the real axis (*Z_r_*). The following formula will be used to compute the DC conductivity (*σ_dc_*) of the sample using the value of *R_b_* and the dimensions of the sample [21]:

(5) $$\sigma_{Dc}=\frac{\left(1\right)}{R_{b}}\times \left(\frac{t}{A}\right)$$

here, *t* denotes the thickness of the of the polymer electrolyte film and *A* denotes the surface area of the film. The values computed for the DC conductivity of each sample are shown in Table 3. One of the key factors to employ a polymer blend electrolyte in practical use such as electrical double-layer capacitors (EDLCs) is having a relatively high DC conductivity. The equation given below shows the dependence of the degree of conductivity of electrolytes on the number density and the mobility of the ions [21,74]:

(6) $$\sigma_{Dc}=\sum \eta qµ$$

where, *η* denotes the carrier density, *q* denotes simple charge and μ represents mobility. Previous research suggested that the charge species of H^+^ ion is released by an ammonium ion in a polymer–ammonium salt system [75]. The DC conductivity of electrolyte samples of CS:PEO: NH_4_BF_4_ (at ambient temperature) is depicted in Table 3. It is clear that the value of *R_b_* decreased only for the samples with low salt concentration (10 to 20 wt.% of NH_4_BF_4_). However, further inclusion of the salt (30 and 40 wt.% of NH_4_BF_4_) clearly increased the value of *R_b_*, which is an important finding of this work. At low salt concentration the value of *R_b_* dropped, because the dissociated salt provides more charge carriers (mobile ions) to the host polymer, which give rise to the DC conductivity (σ) [σ ∝ carrier density] and decrease the (*R_b_*) value. However, at high salt concentration due to the ion association the total number of charge carriers declined and (σ) also decreased, which in turn increased the value of *R_b_*. In addition, at high salt concentration the crystallinity phase of the prepared polymer electrolyte samples increased, which also restricted the movement of mobile ions (amorphous phase act as a pathway for mobile ions) and resulted in larger value of *R_b_*. Each of the XRD, FESEM and EIS results confirmed the above discussion and is in good agreement. 

It has been documented that the gained DC conductivity for PEO proton conducting polymer electrolyte is in the range between 10^−8^ and 10^−7^ S cm^−1^ [76]. Figure 4c,d exposed the DC conductivity decrement when the salt amount is 30 and 40 wt.%. It has also been documented by researchers that it is critical to apply polymer electrolytes with large DC conductivity from 10^−5^ to 10^−3^ S cm^−1^ in electrochemical devices uses for instance as EDLCs and batteries [41,42,43,44]. For this reason, it is possible to say that polymer electrolytes including NH_4_BF_4_ salt are characterized with low DC conductivity, and hence could not be used in applications. The result of low DC conductivity of the present work is well correlated with salts’ lattice energy. The lattice energy of other ammonium salts, such as ammonium chloride (NH_4_Cl) is 698 kJ/mol; while ammonium bromide (NH_4_Br) is 665 kJ/mol [77]. Previous studies [42,78,79] documented that NH_4_SCN (605 kJ/mol) [80] and NH_4_I (634 kJ/mol) [77] salts are essential to be applied in the polymer electrolytes fabrication for device uses. Dissociation of ions could be more easy since the salt lattice energy is low [81]. However, the previous outcomes demonstrated that the device performance decreases with increasing cycle numbers [42,78,79]. This is related to the fact that the salt with low lattice energy is responsible for ion association again at higher cycles. Thus, it is important to use salts with medium lattice energy such as NH_4_NO_3_ (646 kJ/mol) [77]. In this work, NH_4_BF_4_ is used as the ionic source. NH_4_BF_4_ has low lattice energy of 582 kJ/mol [82] which is slightly lower than NH_4_SCN (605 kJ/mol) and NH_4_I (634 kJ/mol) salts [77,80]. The lattice energy of any salt such as ammonium salts used in polymer electrolytes can significantly affect the DC conductivity value of the prepared electrolyte. Table 4 illustrates the relation between the lattice energy of various ammonium salts with their DC connectivity compared to the current work. It clear that decreasing the lattice energy below 600 kJ/mol can substantially decrease the value of DC conductivity as shown in Table 4. The lattice energy (*U_L_*) of NH_4_BF_4_ in this work with some of other ammonium salts have been measured using the Kapustinskii equation given below and tabulated in Table 5 [83]:

(7) $$U_{L}=\frac{1202\left(\upsilon \right)\left(Z^{+})(Z^{-}\right)}{d_{0}}\left(1-\frac{0.345}{d_{0}}\right)$$

where *v* stands for the number ion and *d*_0_ denotes the sum of cations and anions radii. *Z^+^* and *Z^−^* are the charge number. The measured lattice energy of NH_4_BF_4_ using the Kapustinskii equation is 571.141 kJ/mol. The calculated lattice energy of NH_4_BF_4_ in reference [82] is 582 kJ/mol which is very close to the measured lattice energy using Kapustinskii equation in this work. 

It is well known that the anions and cations of the dissolved salts recombine during evaporation of the solvent. This might be connected to the fact that the electrostatic force among functional groups in polymers and cations is smaller than that present amongst anions and cations and as a result further agglomerated ions will emerge on the film surface. From the above discussion we conclude that the knowledge of lattice energy of salts is important to be considered in the fabrication of polymer electrolytes. 

**Figure 5:**
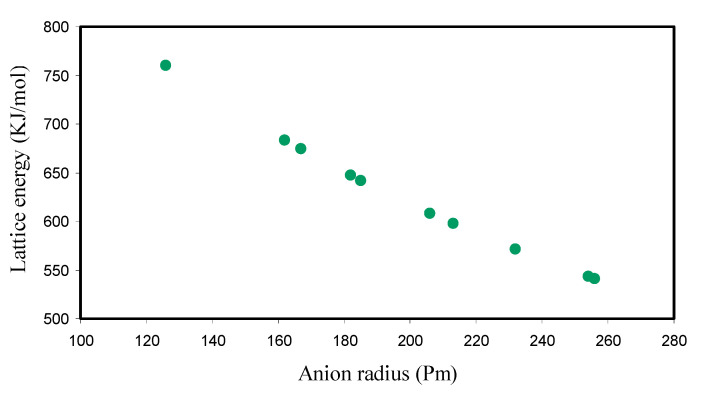
Calculated lattice energy versus anion radius.

Figure 5 shows the lattice energy of some ammonium salts versus anion radius. There are two main important factors that contribute to the lattice energy of salts. One is the charge on the ions, and the other is the radius of the ions. When the ion charge raises, the lattice energy of the salt raises and as the ion radius raises, the lattice energy of the salt reduces (see Figure 5).

### 3.4. Dielectric Properties and Electric Modulus Analysis

**Figure 6:**
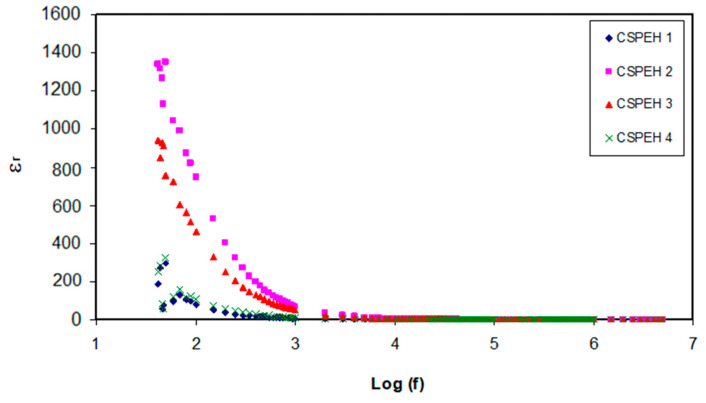
Dielectric constant in opposition to frequency logarithmic scale for the electrolytes.

**Figure 7:**
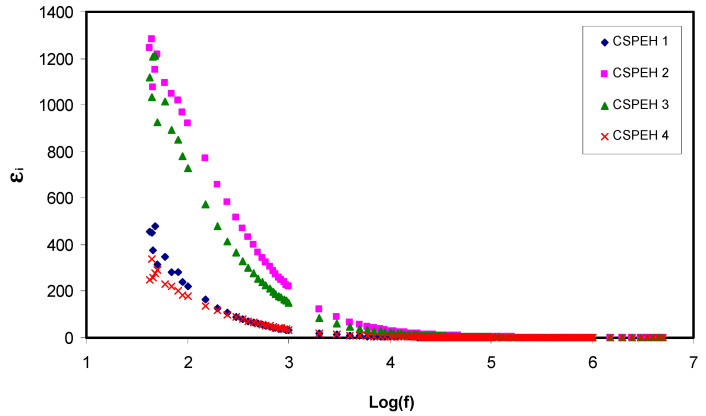
Dielectric loss in opposition to frequency logarithmic scale for all the electrolytes.

The dielectric properties must be calculated in order to analyze the ionic conductivity of the polymer electrolyte. The amount of free mobile ions in the polymer electrolyte can be augmented for the improvement of the conductivity and this can be shown in dielectric spectra [88,89]. This helps in gaining comprehension about polymers as well as their ionic conductivity and crystallinity [90]. The relation of dielectric constant (*ε*′) and dielectric loss (*ε*″) in opposition to frequency can be seen in Figure 6 and Figure 7, respectively. The following expressions have been used to calculate the real and imaginary parts of complex permittivity;

(8) $$\varepsilon^{\prime }=\frac{Z^{\prime \prime }}{\omega C_{o}\left({Z^{\prime }}^{2}+Z^{\prime \prime 2}\right)}$$

(9) $$\varepsilon^{\prime \prime }=\frac{Z^{\prime }}{\omega C_{o}\left({Z^{\prime }}^{2}+Z^{\prime \prime 2}\right)}$$

where *Z*′ and *Z*″ are real part and imaginary part of the complex impedance (Z*), respectively. Clearly, *ε*′ and *ε*″ decline gradually until they attain the lowest value when there is a rise in the frequency and become nearly constant when frequencies are extremely high. The electrode polarization effect is responsible for the higher values acquired for each parameter in the low-frequency region [91,92]. The following relation shows the strong association between dielectric constant (*ε*′) and density number of charge carriers (*n*);

(10) $$n=n_{0}\exp(-U/\varepsilon^{\prime }K_{B}T)$$

where *U* represents the dissociation energy, *T* represents the absolute temperature, *n*_0_ represents pre-exponential constant, and *k_B_* represents the Boltzmann constant. This implies direct proportionality between dielectric constant and DC conductivity. It is also found that (σ = Σ qn_i_µ_i_) with q represents the charge on the ion carriers, which also implies that the charge carrier density (n_i_) and the mobility (µ_i_) are also factors that help to determine DC ionic conductivity of polymer electrolytes [7,74,93]. Thus, dielectric constant is an essential parameter that provides better insight into the electrical properties of polymer electrolytes as well as in the prediction of samples’ conductivity behaviors. This discussion and explanation about the movement of ions and the association between dielectric constant (*ε*′) and DC conductivity highlights the complexity of the ion transport mechanism in polymer electrolyte systems [94]. The main issue that prevents the achievement of a high-conducting polymer electrolyte at ambient temperature is limited knowledge of the transport mechanism of the cation in the polymer electrolytes [95,96]. This suggests that there are other factors besides concentration of ions species and segmental mobility associated with the behavior of the conductivities of polymer electrolytes, including dielectric constant and dissociation energy of ions that are essential for the movement of ions.

### 3.5. Electric Modulus Study

**Figure 8:**
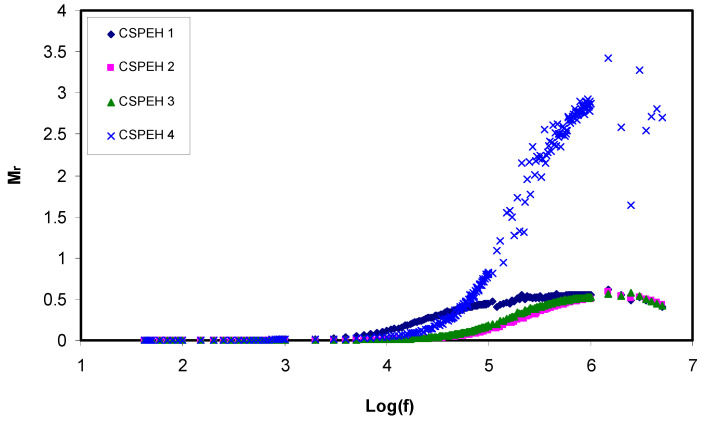
Electric modulus (M_r_) real part in opposition to the frequency logarithmic scale for all the electrolytes.

**Figure 9:**
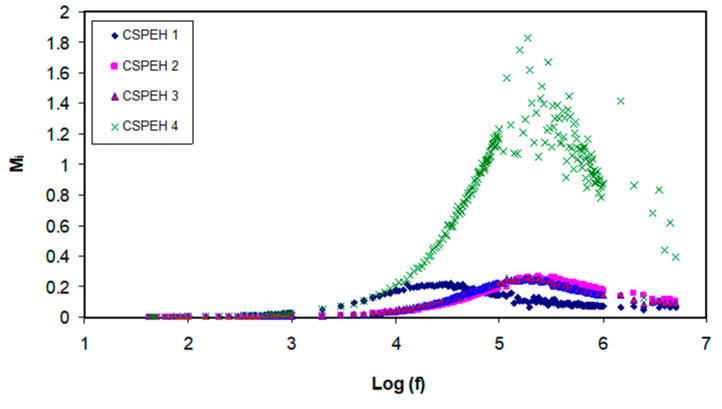
Electric modulus (M_i_) imaginary part in opposition to the frequency logarithmic scale for all the electrolytes.

Electrical modulus formalism can be helpful in conducting further research related to the dielectric performance. The modulus can be easily shown by repressing the signal intensity associated with electrode polarization or by focusing on little details in the high-frequency region [97]. Hence, the conductivity and the relaxation associated with conductivity in ionic conductors as well as polymers can be conveniently studied with the help of the electric modulus curves [8]. The issues that impeded the complete analysis and detailed permittivity relaxation including electrode nature, space charge mechanism and conduction impacts can be addressed [55,92,98,99]. The formulas given below allow the calculation of the real and imaginary parts of complex electric modulus (M*) by inserting the values of the real (*Z_r_*) and imaginary (*Z_i_*) parts of complex impedance (Z*) [55,82]:

(11) $$M^{\prime }=\frac{\varepsilon^{\prime }}{\left({\varepsilon^{\prime }}^{2}+\varepsilon {\prime \prime }^{2}\right)}=\omega C_{o}Z^{\prime \prime }$$

(12) $$M^{\prime \prime }=\frac{\varepsilon \prime \prime }{\left({\varepsilon^{\prime }}^{2}+\varepsilon {\prime \prime }^{2}\right)}=\omega C_{o}Z^{\prime }$$

where angular frequency is denoted by *ω*, capacitance of dielectric cell without the sample is denoted by *C_o_*, *Z_r_* denotes the real part of impedance and *Z_i_* denotes the imaginary part of the impedance. The real and imaginary parts of electric modulus are donated by *M*′ and *M*″, respectively and depicted in Figure 8 and Figure 9. When the frequency is low, a lower value is depicted by both the *M*′ and *M*″ regions, which shows that the electrode polarization effect is suppressed. Conversely, when frequency is high, both real and imaginary electric modulus also augments and depicts clear peaks in the *M*″ spectra. These peaks are referred to as the conduction relaxation peaks. The mobility of the charge carriers over long distance is obvious when the frequencies are lower than peak frequencies (f_max_) [98,99]. There is no similarity in regions beside the maximum peaks which show the absence of the behavior of the ideal Debye-kind. The transfer of the (*M*″) peaks in the direction of the higher frequencies indicates a decline in relaxation time, which consequently boosts the ionic mobility as well as the electrical conductivities; on the contrary, its shift in the left direction indicates a decline in ion mobility and a consequent decline of conductivity [35,100,101,102].

## 4. Conclusions

In conclusion, ammonium salts with low lattice energy below 600 kJ/mol are not desired for electrochemical device applications due to the high tendency of ion association among cations and anions of the dissolved salt. In this work, NH_4_BF_4_ salt as an example of low lattice energy ammonium salt was examined with CS:PEO polymer blends via structural, morphological and electrical characterizations. The increased crystalline region with rising salt concentration was observed in the XRD pattern which can be attributed to the leakage of the associated ions to the surface of the polymer. The outcome from XRD deconvolution analysis demonstrated that the polymer electrolyte with utmost DC ionic conductivity exhibits the smallest X_C_. These results were confirmed by the SEM study. The SEM images showed the protruded salts with a large size at high salt concentration which increased the crystallinity of the prepared samples. The impedance analysis revealed the increment of bulk resistance due to the association of cations and anions. The stumpy value of DC conductivity addressed the non-suitability of the electrolytes in electrochemical device applications. Polymer electrolytes are the kindness of electrochemical devices. Ion transport with high DC conductivity in polymer electrolytes is of great interest. To maximize the availability of an electrolyte system for application it is important to consider the lattice energy of the selected salt. The achieved experimental results in the present work and the calculated lattice energy of salts based on the Kapustinskii equation indicated that salts with moderate lattice energy may be better to consider for reducing the probability of ion association. The electrode polarization phenomena were observed as high value of dielectric constant was recorded at the low-frequency region. The investigation of complex electric module exposed a difference in regions around the maximum peaks which indicated non-Debye type behavior. This implies that there were various polarization mechanisms occurring and many interactions between ions and dipoles exist, while there was a Debye-type relaxation process based on non-interacting identical dipoles.

## Figures and Tables

**Table 1:** The degree of crystallinity from deconvoluted XRD analysis.

Electrolyte	Degree of Crystallinity (%)
CSPEH1	8.52
CSPEH2	7.43
CSPEH3	31.82
CSPEH4	65.84

**Table 2:** The electrical equivalent circuit (EEC) fitting parameters for electrolyte system at surrounding temperature.

Sample	P_1_ (rad)	P_2_ (rad)	*K*_1_ (*F*^−1^)	*K*_2_ (*F*^−1^)	*C*_1_ (*F*)	*C*_2_ (*F*)
CSPEH1	0.8784	0.3946	1.1 × 10^9^	7.6 × 10^5^	9.09 × 10^−10^	1.32 × 10^−6^
CSPEH2	0.8847	0.6072	8.0 × 10^8^	7.0 × 10^5^	1.25 × 10^−9^	1.43 × 10^−6^
CSPEH3	0.8338	0.5410	8.5 × 10^8^	7.10 × 10^5^	1.18 × 10^−9^	1.41 × 10^−6^
CSPEH4	0.7765	0.5665	1.01 × 10^9^	3.20 × 10^6^	9.90 × 10^−10^	3.13 × 10^−7^

**Table 3:** Computed direct current (DC) ionic conductivity for the chitosan:poly(ethylene oxide) (CS:PEO) blend electrolyte sample.

Prepared Films	DC Conductivity (S cm^−1^)
CSPEH1	8.726 × 10^−8^
CSPEH2	7.932 × 10^−7^
CSPEH3	5.817 × 10^−7^
CSPEH4	1.246 × 10^−7^

**Table 4:** Relation between DC ionic conductivity and lattice energy of different ammonium salts.

Ammonium Salts	Lattice Energy (kJ/mol)	DC Conductivity (S cm^−1^)
NH_4_F	759	2.96 × 10^−3^ [30]
NH_4_NO_3_	642	1.6 × 10^−3^ [62]
NH_4_I	608.33	1.12 × 10^−3^ [42]
NH_4_SCN	597.842	1.7 × 10^−3^ [31]
NH_4_BF_4_	571.141	1.246 × 10^−7^ [this work]

**Table 5:** Lattice energy for some of the ammonium salts.

Ammonium Salts	Cation	Anion	Cation Radius (pm) [84]	Anion Radius (pm) [84]	Lattice Energy (kJ/mol)
NH_4_CF_3_SO_3_	NH_4_^+^	CF_3_SO_3_^−^	151	256 [85]	540.59
NH_4_PF_6_	NH_4_^+^	PF_6_^−^	151	254 [86]	543.06
NH_4_BF_4_	NH_4_^+^	BF_4_^−^	151	232	571.141
NH_4_SCN	NH_4_^+^	SCN^−^	151	213 [87]	597.842
NH_4_I	NH_4_^+^	I^−^	151	206	608.33
NH_4_NO_3_	NH_4_^+^	NO_3_^−^	151	185	642
NH_4_Br	NH_4_^+^	Br^−^	151	182	647.13
NH_4_Cl	NH_4_^+^	Cl^−^	151	167	674.02
NH_4_CH_3_COO	NH_4_^+^	CH_3_COO^−^	151	162	683.41
NH_4_F	NH_4_^+^	F^−^	151	126	759.82
